# The 2017 Dengue virus 1 outbreak in northern Vietnam was caused by a locally circulating virus group

**DOI:** 10.1186/s41182-021-00386-0

**Published:** 2022-01-04

**Authors:** Taichiro Takemura, Co Thach Nguyen, Ha Chau Pham, Thuy Trang Nguyen, Vu Mai Phuong Hoang, Le Khanh Hang Nguyen, Takeshi Nabeshima, Thi Thu Thuy Nguyen, Thi Quynh Mai Le, Meng Ling Moi, Kouichi Morita, Futoshi Hasebe

**Affiliations:** 1 Vietnam Research Station, Institute of Tropical Medicine, Nagasaki University, 1-12-4 Sakamoto, Nagasaki-shi, Nagasaki, Japan; 2 Department of Virology, Institute of Tropical Medicine, Nagasaki University, Nagasaki, Japan; 3 Graduate School of Biomedical Sciences, Nagasaki University, Nagasaki, Japan; 4 Program for Nurturing Global Leaders in Tropical and Emerging Communicable Diseases, Nagasaki University, Nagasaki, Japan; 5 Department of Virology, National Institute of Hygiene and Epidemiology, Hanoi, Vietnam; 6 School of International Health, Graduate School of Medicine, University of Tokyo, Tokyo, Japan

**Keywords:** Dengue virus, Vietnam, Viral genome, Virus circulation

## Abstract

**Background:**

Dengue virus (DENV) is a member of insect vector-borne viruses, and it causes dengue fever. Southeast Asia is the epi-center of dengue fever in the world. The characterization of the virus is essential to identify the transmission and evolution of DENV.

**Objectives:**

In 2017, there was an outbreak of Dengue virus type 1 (DENV1) in northern Vietnam and the neighboring countries. To identify the genetic character of the outbreak virus in the area, we conducted whole-genome sequencing analysis on the samples positive for the DENV1 along with real-time PCR.

**Study design:**

In total, 1026 blood samples were collected from patients with suspected dengue fever in Ha Nam and Hai Duong province, nearby areas of the capital of Vietnam. After screening by real-time PCR, 40 of DENV1 positive samples were subjected to whole-genome sequencing, and 28 complete coding sequences were obtained.

**Results:**

All 28 sequences were genotype I of DENV1, which is dominant in the southeast and East Asian countries. The phylogenetic analysis of the E region showed that they fell into a single cluster with the reported sequences from Vietnam between 2009 and 2016, in which the isolates from other countries are very rare. Our results suggested that the 2017 outbreak in the area was caused by locally circulating viruses.

**Supplementary Information:**

The online version contains supplementary material available at 10.1186/s41182-021-00386-0.

## Background

Dengue virus (DENV) is the causative agent of dengue fever and dengue hemolytic fever or dengue shock syndrome. Four different serotypes (DENV1–4) have been identified and are known to be transmitted by two mosquito species, *Aedes aegypti* and *A. albopictus* [1]. Dengue fever is a major global health threat as it affects more than 100 countries worldwide. In the summer of 2017, we experienced a large-scale outbreak of DENV1 infection in northern Vietnam [2]. According to the World Health Organization situation report, 36,345 DENV1 cases had been recorded in Hanoi from January through November; and this number was approximately 4–5 times higher than that of the previous year [3]. The DENV1 outbreak in 2017 was also observed in the neighboring countries. In Xishuangbanna, located along the southern border of China, over 1,100 cases were confirmed during the same period [4].

In this study, we aimed to elucidate the genetic characteristics of the DENV1 strain that caused the 2017 outbreak. To this end, we conducted a whole-genome analysis of DENV1 on serum samples collected from the patients in Ha Nam and Hai Duong, which are located in northern Vietnam.

## Methods

Blood samples were collected from patients suspected to have dengue fever, who visited Ha Nam Province General Hospital and Hai Duong Preventive Medicine Center. Among 1026 tested samples, 252 were positive for DENV, determined by real-time PCR (RT-PCR), including 227 cases of DENV1, 23 cases of DENV2, two cases of DENV3, and two cases of DENV4 [5, 6]. Two samples were dual positive for DENV1 and DENV2. Viral RNA was extracted using a Qiagen viral RNA mini kit (Qiagen, Aarhus, Denmark) from each serum sample to carry out a whole-genome analysis of DENV1. Superscript III (Life Technologies, CA, USA) was employed to synthesize cDNA, using random hexamers, and the cDNA was subjected to library preparation using a NExtera XT kit (Illumina, San Diego, CA, USA). Sequences were obtained by Miseq (Illumina) [7]. The raw reads were mapped to the reference DENV1 sequence (EU848545), and the consensus sequences were obtained using a CLC Genome Workbench (Qiagen).

Anti-dengue IgM and IgG detection was performed on the samples available for the whole genome sequences using an ELISA kit (Dengue ELISA IgM CAPTURE and Dengue ELISA IgG, VIRCELL, Granada, Spain) to determine the infection status (primary or secondary). Whether a sample was positive or negative was judged according to the manufacturer’s instructions. Since the DENV1 infection was confirmed by serotype-specific RT-PCR, we defined the infection status as follows: Primary infection was defined as IgG negative and IgM positive or IgG and IgM negative; secondary infection; IgG positive.

## Results

**Fig. 1:**
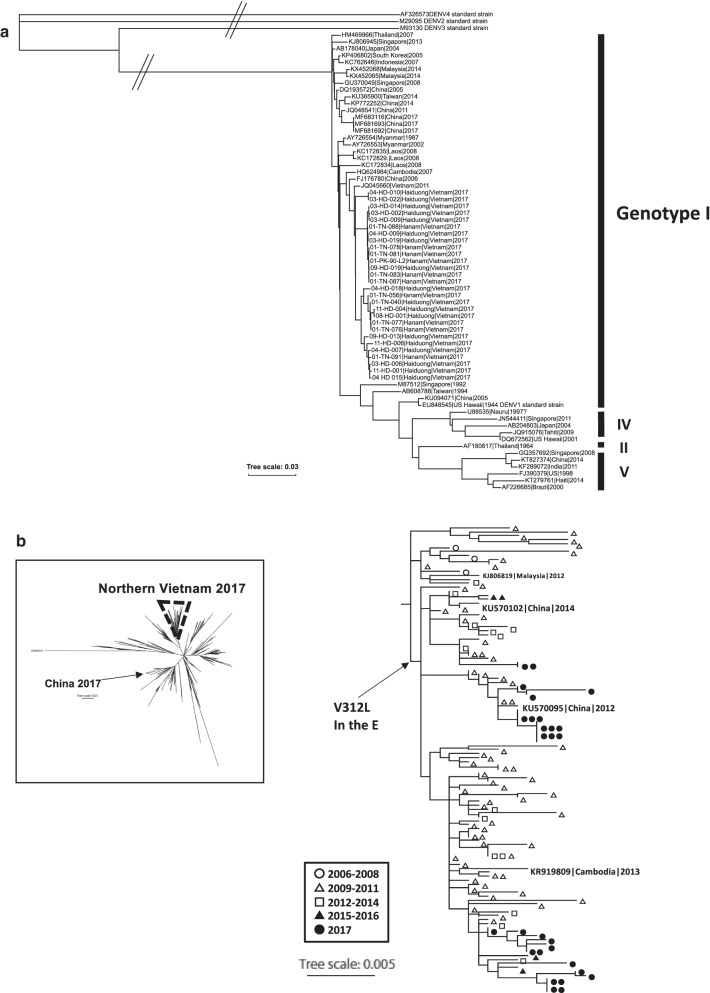
**a** Phylogenetic tree constructed by the whole genome sequence together with DENV1-4 standard strains and genotype I, II, IV and V sequences. The sample lists are attached to the supplementary material. The accession numbers of newly defined samples are also described in supplementary materials. **b** Expanded phylogenetic tree constructed by the complete E gene sequence together with genotype I isolates recorded in the ViPR database from 6 southeast or East Asian countries. Open circle: the Vietnames isolates between 2006 and 2008; Open triangle: between 2009 and 2011; Open square: between 2012 and 2014: black triangle: between 2015 and 2016: black circle: 2017, analyzed in this study

We succeeded in obtaining 28 complete coding sequences from 40 patients with DENV1 infection with more than 30 coverage. All of the newly identified DENV1 sequences from northern Vietnam fell into the genotype I cluster, which is dominant in southeastern and eastern Asia (Fig. 1a). This cluster could be separated into four subclusters and was close to the JQ045660 isolate obtained in Vietnam in 2011. Notably, these isolates were phylogenetically distinct from the strains identified in China during the same period (MF681693, MF683116, and MF681692) [4], suggesting that the origin of DENV1 in northern Vietnam was different from the isolates found in nearby areas. We identified 135 amino acid substitutions in comparison with the standard genotype 1 strain, EU848545, and nine substitutions were conserved in all newly sequenced samples. Two substitutions are in the structural region, A173T in precursor membrane (PrM), and V312L in the Envelope protein (E). Seven of these substitutions were in the nonstructural region, including one in the Nonstructural protein 1 (NS1) (H111Y), one in NS2A (K218R), one in NS4B (T27A), and four in NS5 (R378K, K551R, V787I, and E833G) (Additional file 1: Table S1).

Available full-length genome sequences of DENV1 are insufficient for detailed analyses, therefore we analyzed the E gene region (approximately 1483 bp) together with reference sequences obtained from the Virus Pathogen Database and Analysis Resource (ViPR, NCBI) before the year of 2017 [8]. In total, we analyzed 1215 sequences from isolates obtained from southeastern and eastern Asia (287 from China, 261 from Thailand, 98 from Cambodia, 90 from Laos, 132 from Myanmar, 75 from Malaysia, and 272 from Vietnam; Additional file 2: Table S2). We aligned all the sequences using MEGA7 and then constructed a phylogenetic tree using the maximum likelihood (ML) method with 100 bootstraps [9]. Consistent with the whole genome analysis results, the 2017 Northern Vietnamese sequences fell into a single cluster with four closely related subgroups (Fig. 1b). The cluster included Vietnam isolates obtained in 2008 and 2016, in addition to two sequences from China (2012 and 2014), and one from Cambodia (2013). Chinese isolates in 2017 formed a distinct cluster with other Chinese, Malaysian, and Vietnamese isolates, which suggested that the group of DENV1 causing the large-scale outbreak in 2017 was circulating locally in the area. The cluster containing 2017 isolates encoded the V312L substitutions in the E region. On the database, the V312L substitution did not find any other group of DENV1.

The analysis of IgM and IgG indicated that 13 samples were categorized as a primary infection, and 13 samples as secondary infection (2 samples were not available because of the small sample volume) (Additional file 3: Table S3). For the phylogenetical analysis and amino acid sequence comparison, we did not observe conserved substitutions according to the infection status.

## Discussions

The DENV1 sequences collected during 2017 outbreak period fell into a single cluster of genotype I with the V312L substitution in the E region. The cluster was consisted with the reported sequence from Vietnam after 2009 with a few exceptions from China and Cambodia. The V312L substitution was not found any other DENV1 sequence record on the database so far, it is suggested that the DENV1 variant causing for the 2017 outbreak was locally circulating variant. In Northern Vietnam, large scale DENV1 outbreak is recorded in 2009 [11, 12], and phylogenetic analysis showed high diversity in the 2009 isolates. A possible scenario is that part of the expanded variant encoding V312L in 2009 circulated and maintained within the region, and caused the outbreak in 2017.

Dengue incidence has been increasing over time globally. In Vietnam, the disease was reported to spread to South and Central regions but was not frequent in Northern and Highland regions until 1996. However, seasonal endemics were observed in Hanoi, northern region, after 1997, and large-scale outbreaks were also reported every 3–5 years. Such expansion of DENV infection is considered to be related to the environmental conditions and human mobility according to the social development of the affected countries. Since further civilization cannot be stopped, the risk of dengue will increase globally. Identification of the trigger of the outbreak may lead to the early prevention from DENV infection. Various viral and nonviral factors should be analyzed in the affected area [10]. Although such information was limited in the present study, we concluded that the DENV1-induced outbreak in 2017 in Vietnam was caused by locally circulating viruses. Elimination of locally circulating viruses is more challenging than the prevention of new viral invasion. The development of effective vaccines, treatments, and outbreak prediction tools is urgently needed to protect humans from DENV.

## Conclusions

Our results suggested that the 2017 outbreak in the area was caused by locally circulating viruses. Control of locally circulating viruses is more complicated than preventing new viral invasion; however, the need to develop useful tools to reduce the risk of DENV infection is urgent.

## Supplementary Information

**Additional file 1: Table S1.** Amino acid substitutions detected in 2017 DENV1 viruses. Substitutions are indicated on the location of DENV1 standard strain EU848545.

**Additional file 2:** **Table S2.** List of sequence data used for phylogenetic analysis. The accession numbers of the 28 newly sequenced DENV1 are also provided in the Table.

**Additional file 3: Table S3.** The results of IgG and IgM detection by ELISA kit.

## References

[1] World Health Organization. Dengue: guidelines for diagnosis, treatment, prevention and control, WHO Press. (2009). 23762963

[2] Huy BV, Hoa LNM, Thuy DTV, Kinh N, Ngan TTD, Duyet LV, Hung NT, Minh NNQ, Truong NT, Chau NVVE (2017). Epidemiological and Clinical Features of Dengue Infection in Adults in the 2017 Outbreak in Vietnam. Biomed Res Int.

[3] Dengue Situation Update Number 529. apps.who.int/iris/bitstream/handle/10665/274099/Dengue-20171107.pdf?sequence=4&isAllowed=y Accessed 18 Sept 2018.

[4] Wen S, Ma D, Lin Y, Li L, Hong S, Li X, Wang X, Xi J, Qiu L, Pan Y, Chen J, Shan X, Sun Q (2018). Complete genome characterization of the 2017 Dengue outbreak in Xishuangbanna, a border city of China Burma and Laos. Front Cell Infect Microbiol.

[5] Johnson BW, Russell BJ, Lanciotti RS (2005). Serotype-Specific Detection of Dengue Viruses in a Fourplex Real-Time Reverse Transcriptase PCR Assay. J Clin Microbiol.

[6] Grywna K, Kupfer B, Panning M, Drexler JF, Emmerich P, Drosten C, Kümmerer BM (2010). Detection of all species of the genus Alphavirus by reverse transcription-PCR with diagnostic sensitivity. J Clin Microbiol.

[7] Batovskaa J, Lyncha SE, Rodonia BC, Sawbridgea TI, Cogan NO (2017). Metagenomic arbovirus detection using MinION nanopore sequencing. J Virol Methods.

[8] Pickett BE, Sadat EL, Zhang Y, Noronha JM, Squires RB, Hunt V, Liu M, Kumar S, Zaremba S, Gu Z, Zhou L, Larson CN, Dietrich J, Klem EB, Scheuermann RH (2012). ViPR: an open bioinformatics database and analysis resource for virology research. Nucleic Acids Res.

[9] Kumar S, Stecher G, Tamura K (2016). MEGA7: Molecular Evolutionary Genetics Analysis version 7.0 for bigger datasets. Mol Biol Evol.

[10] Pang T, Mak TP, Gubler DJ (2017). Prevention and control of dengue—the light at the end of the tunnel. Lancet Infect Dis.

[11] Cheng J, Bambrick H, Yakob L, Devine G, Frentiu FD, Toan DTT, Thai PQ, Xu Z, Hu W. Heatwaves and dengue outbreaks in Hanoi, Vietnam: New evidence on early warning. PLoS Negl Trop Dis. 2020;14(1): e0007997. 10.1371/journal.pntd.0007997 PMC6994101 31961869

[12] Dang TT, Pham MH, Bui HV, Van Le D (2020). Whole genome sequencing and genetic variations in several dengue virus type 1 strains from unusual dengue epidemic of 2017 in Vietnam. Virol J.

